# Supplementary material for: The Effect of High Pressure Homogenization on the Structure of Dual-Protein and Its Emulsion Functional Properties
Source: Foods. 2023 Sep 7;12(18):3358. doi: 10.3390/foods12183358 (PMC10529657; doi:10.3390/foods12183358)
Supplement: Supplementary file 1 [file foods-12-03358-s001.zip › foods-2567196-supplementary.pdf]

S1

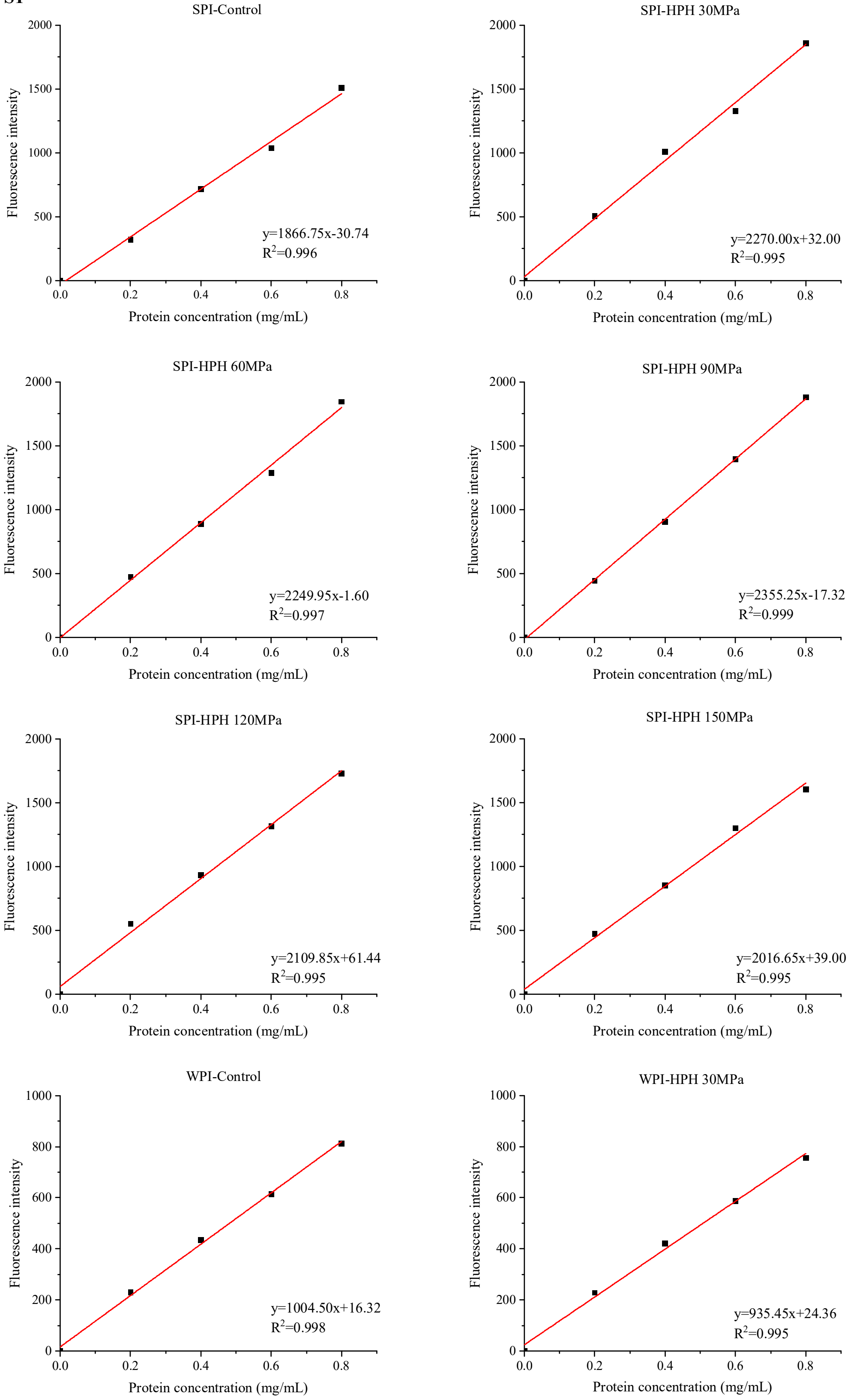
SPI-Control
y=1866.75x-30.74
R^2=0.996
SPI-HPH 30MPa
y=2270.00x+32.00
R^2=0.995
SPI-HPH 60MPa
y=2249.95x-1.60
R^2=0.997
SPI-HPH 90MPa
y=2355.25x-17.32
R^2=0.999
SPI-HPH 120MPa
y=2109.85x+61.44
R^2=0.995
SPI-HPH 150MPa
y=2016.65x+39.00
R^2=0.995
WPI-Control
y=1004.50x+16.32
R^2=0.998
WPI-HPH 30MPa
y=935.45x+24.36
R^2=0.995
Fluorescence intensity
Protein concentration (mg/mL)

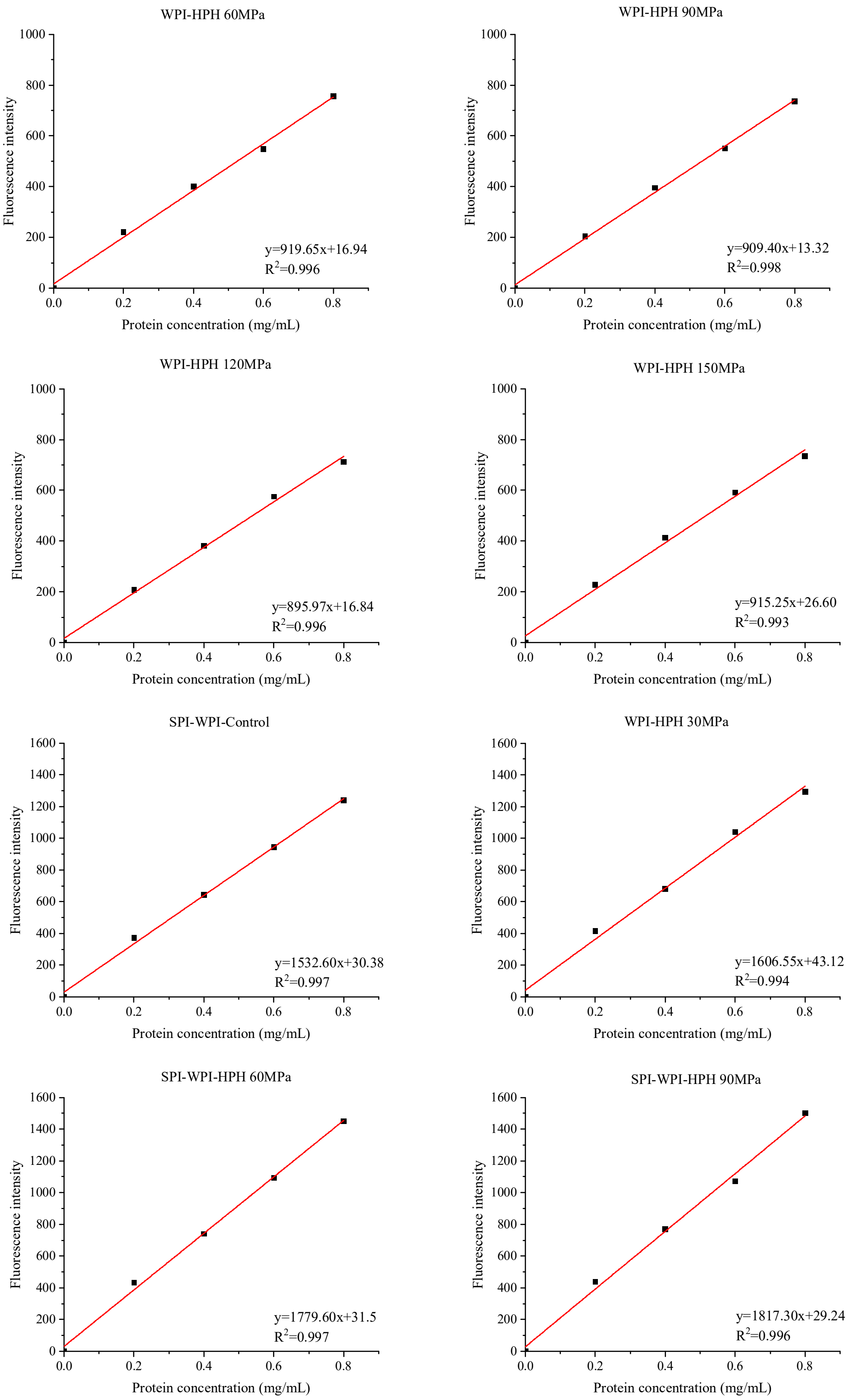
WPI-HPH 60MPa
y=919.65x+16.94
R^2=0.996
WPI-HPH 90MPa
y=909.40x+13.32
R^2=0.998
WPI-HPH 120MPa
y=895.97x+16.84
R^2=0.996
WPI-HPH 150MPa
y=915.25x+26.60
R^2=0.993
SPI-WPI-Control
y=1532.60x+30.38
R^2=0.997
WPI-HPH 30MPa
y=1606.55x+43.12
R^2=0.994
SPI-WPI-HPH 60MPa
y=1779.60x+31.5
R^2=0.997
SPI-WPI-HPH 90MPa
y=1817.30x+29.24
R^2=0.996
Fluorescence intensity
Protein concentration (mg/mL)

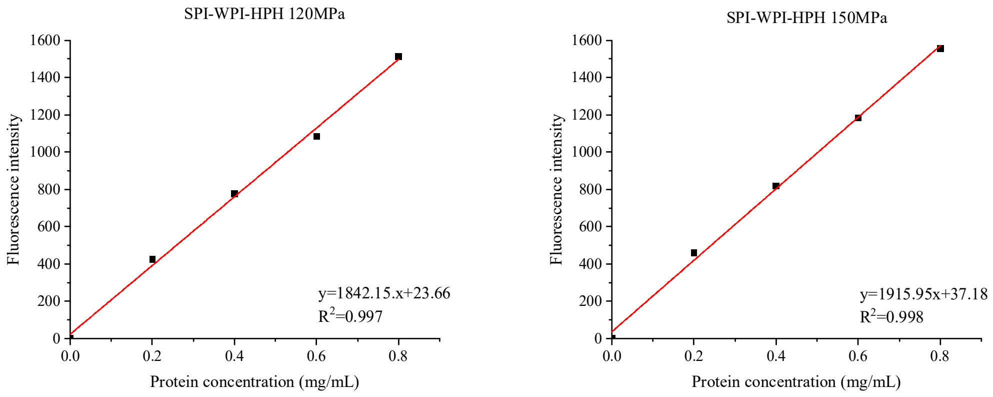

Figure S1. The surface hydrophobicity index (H_0) of SPI, WPI, SPI-WPI. The slope of each curve indicates the H_0 of protein.
